# Long-term survival of participants in the PASART-1 and PASART-2 trials of neo-adjuvant pazopanib and radiotherapy in soft tissue sarcoma

**DOI:** 10.2340/1651-226X.2025.42333

**Published:** 2025-01-15

**Authors:** Bauke H. G. van Riet, Milan van Meekeren, Marta Fiocco, Aisha B. Miah, Ilse de Pree, Lisette M. Wiltink, Astrid Scholten, Lotte Heimans, Judith V. M. G. Bovee, Hans Gelderblom, Neeltje Steeghs, Rick L. Haas

**Affiliations:** a Department of Radiotherapy, the Netherlands Cancer Institute, Amsterdam, the Netherlands; b Department of Medical Oncology, Leiden University Medical Center, Leiden, the Netherlands; c Department of Biomedical Data Science, section Medical Statistics, Leiden University Medical Center, Leiden, the Netherlands; d Mathematical Institute, Leiden University, Leiden, the Netherlands; e Princess Máxima Center for Pediatric Oncology, Utrecht, the Netherlands; f Department of Clinical Oncology, The Royal Marsden Hospital and The Institute of Cancer Research, London, United Kingdom; g Department of Medical Oncology, Erasmus University Medical Center, Rotterdam, the Netherlands; h Department of Radiotherapy, Leiden University Medical Center, Leiden, the Netherlands; i Department of Medical Oncology, The Netherlands Cancer Institute, Amsterdam, the Netherlands; j Department of Pathology, Leiden University Medical Center, Leiden, the Netherlands; k Department of Medical Oncology, University Medical Center Utrecht, Utrecht, the Netherlands

**Keywords:** Pre-operative, chemoradiotherapy, soft-tissue sarcoma, pazopanib

## Abstract

**Objective:**

This study aims to assess the long-term safety and efficacy of adding pazopanib to neo-adjuvant radiotherapy followed by surgery in patients with high-risk non-metastatic soft tissue sarcoma of the trunk and extremities treated in the PASART-1 and PASART-2 trials, as well as to compare the PASART cohorts to a control cohort receiving standard treatment during the same time period from the Netherlands Cancer Registry (IKNL) to investigate if adding pazopanib improves Overall Survival (OS).

**Methods:**

Updated follow-up data on disease control, survival and long-term toxicities of the PASART-trials were extracted from electronic patient records. The effect of adding pazopanib to neo-adjuvant radiotherapy on OS was investigated by comparing the combined PASART cohorts to the IKNL cohort via direct comparison and exact matching analysis.

**Results:**

PASART-trials included 34 patients, IKNL cohort included 487 patients. After a median follow-up of 75.4 months (range: 30–131 months) the 1-year, 2-year and 5-year OS in the PASART-trials were 97% (95% confidence interval [CI]: 91.5–100), 85.3% (95% CI: 74.2–98.1), 79.3% (95% CI: 66.8–94.2), respectively. Matching resulted in 23 PASART and 89 IKNL patients. Adding pazopanib did not significantly improve OS when compared to standard treatment (IKNL) in a direct comparison (hazard ratio [HR]: 0.58; 95% CI: 0.30–1.13) or matched analysis (HR: 0.70; 95% CI: 0.29–1.73). Long-term toxicities, mainly fibrosis (*n* = 6) and edema (*n* = 2), were observed in 11 PASART patients and comparable to historical controls.

**Interpretation:**

The addition of pazopanib had tolerable long-term toxicity but did not improve OS when compared to a control cohort receiving standard treatment.

## Introduction

Soft tissue sarcoma (STS) is a rare disease that accounts for approximately 1% of all diagnosed cancers. It is a heterogeneous group of malignancies with a presumed mesenchymal cell of origin and that can arise almost anywhere in the body [1]. Risk assessment of localised disease is based on multiple factors, including histological subtype, grade, size and depth of the tumour [2]. In case of high-risk STS, surgery with radiotherapy is the standard treatment, with radiotherapy preferably administered neo-adjuvant due to its favourable late toxicity profile [3]. This combined treatment achieves loco regional tumour control in the vast majority of patients.

Nevertheless, roughly equal to or less than 15% of all STS patients may develop local recurrences and roughly 30% or more may develop distant metastases, which ultimately result in death [4]. One of the potential strategies to improve survival may be to enhance the efficacy of radiotherapy. Therefore, several attempts to find suitable systemic agents and radiosensitisers to enhance radiation efficacy in localised STS have been underway [5]. One of these efforts was the phase I PASART-1 trial (NCT01985295) [6] and phase II PASART-2 trial (NCT02575066) [7], which assessed the addition of the anti-angiogenic pazopanib to the combined modality treatment of neo-adjuvant radiotherapy and surgery in high-risk localised STS.

In both trials, the addition of pazopanib was generally well tolerated. However, both trials observed a high proportion of patients with transient asymptomatic and elevated transaminases. The PASART-2 failed to meet its primary endpoint of a ≥ 30% pathological complete response (pCR) rate (defined as ≤5% viable tumour cells), with an observed pCR rate of 20%. Although the predetermined efficacy endpoint was not met, the observed pCR rate of 20% still exceeds twice the historical pCR rates following standard neo-adjuvant radiotherapy in STS [8].

Thus, while the PASART-trials showed that neo-adjuvant pazopanib combined with radiotherapy appears tolerable, with the exception of asymptomatic, transient elevated transaminases, it also exhibits promising efficacy in terms of achieving pCRs. This increased pCR efficacy should ultimately result in improved long-term outcomes. Therefore, this study reports the long-term follow-up of the combined PASART-1 and PASART-2 cohorts to evaluate the long-term disease control of neo-adjuvant radiotherapy and pazopanib. Furthermore, to study the effect of this pazopanib-based regimen on oncological outcomes, the PASART cohorts are compared to a similar cohort of patients receiving neo-adjuvant radiotherapy without systemic therapies from the Netherlands Cancer Registry of the Netherlands Comprehensive Cancer Organisation (IKNL).

## Methods

### Study design and participants

The study populations of this study were previously published [6, 7, 9]. Briefly, patients of the PASART-trials and IKNL cohort were ≥ 18 years old and had newly diagnosed, high-risk (either deep seated and/or > 5 cm and/or anticipated close resection margin and/or Fédération Nationale des Centres de Lutte Contre Le Cancer [FNCLCC] grade II/III) STS. Patients enrolled in the PASART-trials also received neo-adjuvant radiotherapy and concurrent pazopanib starting 1 week before radiotherapy and continuing until completion. None of the included patients received any additional neo-adjuvant or adjuvant chemotherapy.

In the PASART-1, 11 patients received 50 Gy in 25 fractions between 2011 and 2014. Pazopanib was administered, in three dose levels: 400 mg once daily (*n* = 3), 600 mg once daily (*n* = 4) and 800 mg once daily (*n* = 4). Surgery was performed 5–7 weeks following radiotherapy.

In the PASART-2, the first 21 patients were treated with 50 Gy in 25 fractions between 2016 and 2018. After a protocol amendment, four additional patients were treated with 36 Gy in 18 fractions. All patients received 800 mg pazopanib once daily. Surgery was performed 4–8 weeks following radiotherapy.

The IKNL cohort consists of 2,165 patients diagnosed and treated between 2011 and 2017. It includes 544 patients receiving neo-adjuvant radiotherapy. The precise dose of radiotherapy and the time between the start of treatment and surgery is unknown, but is expected to be according to the then-standard protocols and practices, that is, 50 Gy in 25 fractions followed by surgery 6–8 weeks after radiotherapy.

To create a homogenous group of patients between the PASART-trials and IKNL cohort, IKNL patients who received additional systemic therapy, additional adjuvant radiotherapy or had a sarcoma outside of the trunk and extremities were excluded.

### Data collection

The principal investigator provided the original databases of the PASART-trials and IKNL. Both databases contain patient’s characteristics, treatment and survival data. Unfortunately, disease control data, precise tumour dimensions and pCR status are not available for the IKNL cohort. All PASART patients consented the collection of long-term follow-up data. Updated follow-up data on disease control, survival, and long-term toxicities were extracted from electronic patient files for all patients of PASART-trials. Long-term toxicities were defined as Common Terminology Criteria for Adverse Events (CTCAE) 2.0 grade ≥ 2 toxicities that occurred or persisted ≥ 4 weeks after surgery. Postoperative wound complications were excluded, as these were reported in the primary publications.

Following data collection, patients were staged using the then current American Joint Committee on Cancer (AJCC) STS criteria (7th edition) [10]. Histological subtypes were classified according to the then current 2013 WHO STS classification [11]. Liposarcomas were divided into subtypes due to the difference in clinical behaviour and the known sensitivity of myxoid liposarcoma to radiation [12, 13]. Undifferentiated pleomorphic, spindle cell, and not otherwise specified sarcomas were combined into undifferentiated soft tissue sarcoma (USTS), as there is no clinical distinction between these subtypes, and they have been combined into the broader USTS category in the 2020 WHO STS classification [14]. Age was divided into three categories: < 50 years (young), 50–65 years (middle-aged) and ≥ 65 years (aged). Tumour sizes were divided as < 5 cm and ≥ 5 cm.

### Matching

Since patients in the PASART-trials were not randomised, and there is a possibility of heterogeneity between the PASART and IKNL cohorts due to patient characteristics (primarily histological subtypes), we perform a matched data analysis using exact matching without replacement to account for known confounders on overall survival (OS) in addition to directly comparing both cohorts. Confounders were chosen a priori and included age, sex, AJCC-stage (tumour size, tumour depth and FNCLCC grade), histological subtype, location, and resection margin. Patients with missing data were excluded before matching. Exact matching was chosen over other matching methods, as it is the most straightforward method. Here each case is matched with all possible controls that have the same values for all covariates. To assess matching accuracy and balance, the standardised mean differences of matching variables were calculated. Standardised mean differences of < 0.1 were considered balanced. Alternative matching strategies were conducted to assess sensitivity. Matching was performed using the MatchIt R-package version 4.5.5 for matching and Cobalt R-package version 4.5.3 for determining post-matching variable balance.

### Statistical analysis

Summary tables for continuous variables include median and interquartile range; for categorical variables sample size (*N*) and proportion are reported. Disease-free survival (DFS) was defined as time between surgery and signs of local recurrence or metastasis by either clinical examination or radiological imaging. OS was defined as time between surgery and death from any cause. Patients still alive at the end of follow-up were censored at the last date of contact. OS and DFS were estimated using Kaplan-Meier’s methodology. Log-rank tests were used to assess difference in survival between PASART and IKNL patients. Median follow-up was estimated with the reverse Kaplan-Meier method [15]. Univariate Cox regression models were estimated to study the association between prognostic factors (sex, age, tumour size, tumour depth, FNCLCC grade, AJCC-stage, tumour subtype, tumour location, resection margin and pazopanib treatment) and OS. Cox regression outcomes were reported as hazard ratio (HR) with a 95% confidence interval (CI). Survival outcomes were estimated for each variable. All analyses were performed in R software environment version 4.2.1, library survival version 3.3.1 was used to estimate Cox regression models, Library survminer version 0.4.9 was used to estimate the survival function [16]. *P*-values of < 0.05 were considered significant.

## Results

PASART-1 and PASART-2 cohorts consisted of 11 and 25 patients, respectively. After excluding patients who were reclassified as bone sarcomas (*n* = 1) or did not undergo surgery (*n* = 1), 34 PASART patients remained. The IKNL cohort consisted of 544 patients who received neo-adjuvant radiotherapy and surgery, after excluding patients who had systemic therapy (*n* = 41) or had a tumour localisation outside of the extremities or trunk (*n* = 16), 487 patients remained. Baseline patient and tumour characteristics are described in Table 1.

**Table 1:** Patients’ characteristics.

	Overall		Matched	
	PASART, (*n* = 34)	IKNL, (*n* = 487)	PASART, (*n* = 23)	IKNL, (*n* = 89)
Sex, *n* (%)				
Male	21 (62)	284 (58)	14 (61)	60 (67)
Female	13 (38)	203 (42)	9 (39)	29 (33)
Age at diagnosis (years), median (IQR)	58 (48–67)	62 (49–72)	58 (50–69)	66 (59–75)
Age in groups, *n* (%)				
< 50 years	10 (29)	124 (25)	6 (26)	7 (8)
≥ 50 – > 65 years	14 (41)	148 (30)	9 (39)	36 (40)
≥ 65 years	10 (29)	215 (44)	8 (35)	46 (51)
AJCC-stage, *n* (%)				
Stage I	3 (8.8)	-	-	-
Stage II	18 (53)	231 (47)	16 (70)	43 (48)
Stage III	13 (38)	251 (52)	7 (30)	46 (52)
Unknown	-	5 (1)	-	-
Tumour size, *n* (%)				
< 5 cm	4 (12)	65 (13)	4 (17)	22 (25)
≥ 5 cm	30 (88)	417 (86)	19 (83)	67 (75)
Unknown	-	5 (1)	-	-
Tumour depth, *n* (%)				
Deep	30 (88)	209 (43)	19 (83)	32 (36)
Superficial	4 (12)	180 (37)	4 (17)	38 (42)
Unknown	-	98 (20)	-	19 (21)
FNCLCC grade, *n* (%)				
Grade I	3 (8.8)	-	-	-
Grade II	17 (50)	192 (39)	15 (65)	26 (29)
Grade III	14 (41)	295 (61)	8 (35)	63 (71)
Histological subtype, *n* (%)				
USTS	15 (44)	130 (27)	14 (61)	58 (65)
Myxofibrosarcoma	9 (26)	84 (17)	5 (22)	26 (29)
MPNST	2 (6)	20 (4)	1 (4)	3 (3)
Synovial sarcoma	2 (6)	19 (4)	2 (9)	1 (1)
PLS	1 (3)	19 (4)	1 (4)	1 (1)
MLS	1 (3)	110 (23)	-	-
RMS	2 (3)	3 (1)	-	-
Epithelioid sarcoma	1 (3)	5 (1)	-	-
Clear cell sarcoma	1 (3)	-	-	-
DDLPS	-	26 (5)	-	-
Liposarcoma NOS	-	7 (1)	-	-
LMS	-	33 (7)	-	-
Other/Unknown sarcoma	-	31 (6)	-	-
Tumour site, *n* (%)				
Extremity	26 (76)	388 (80)	20 (87)	82 (92)
Trunk	8 (24)	99 (20)	3 (13)	7 (8)
Resection margin, *n* (%)				
R0	32 (94)	369 (76)	23 (100)	89 (100)
R1	2 (6)	67 (14)	-	-
R2	-	5 (1)	-	-
Unknown	-	46 (9)	-	-
pCr rate, *n* (%)	8 (24)	-	4 (17)	-

AJCC: American Joint Committee on Cancer; DDLPS: dedifferentiated liposarcoma; FNCLCC: Fédération Nationale des Centres de Lutte Contre le Cancer; IQR: interquartile range; pCR: pathologic complete response; MPNST: malignant peripheral nerve sheath tumour; MLS: myxoid liposarcoma; USTS: undifferentiated soft tissue sarcoma; PLS: pleomorphic liposarcoma; RMS: rhabdomyosarcoma; LMS: leiomyosarcoma (excluding skin).

### Long-term follow-up PASART-trials

**Figure 1:**
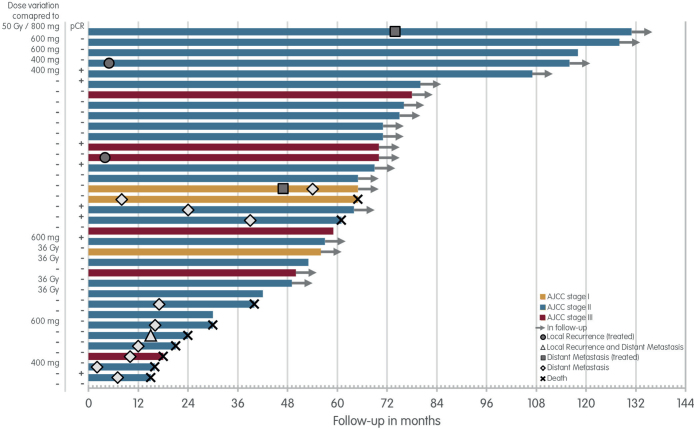
Long-term follow-up PASART-1 and PASART-2. Swimmer’s plot for patient of the PASART-1 and PASART-2 trials, stratified by the AJCC-stage. Dose alterations compared to the most common dose of 800 mg pazopanib and 50 Gy neo-adjuvant radiotherapy and pCR status of the resection specimen are next to each lane. AJCC: American Joint Committee on Cancer stage; Gy: Gray; pCR: pathological complete response.

The follow-up and events of the PASART patients are visualised in Figure 1. The median follow-up of the PASART patients is 75.4 months (range: 30–131 months), PASART-1 92.8 months (range: 30–131 months), and PASART-2 66.3 months (range: 42–80 months). Twenty of the 34 patients are still in follow-up, one completed follow-up, four were lost during follow-up and nine have died.

### Long-term toxicities

Eleven PASART patients (32%) developed long-term toxicities (≥ 4 weeks after surgery). Most of the non-persistent long-term toxicities were already reported in the previous PASART articles, which include four grade 1–2 and one grade 3 fibrosis, two grade 1–2 localised oedema; two grade 1–2 joint range of motion decreased (6, 7). Three new long-term toxicities were identified: one persistent seroma grade 2 that was complicated by infection, one myalgia (leg cramp) grade 1, and one fibrosis grade 1 with skin hyperpigmentation.

### Local recurrence and management

Three PASART patients (9%) developed local recurrences. Two of these developed a local recurrence after a R1 resection at 4 and 5 months after surgery, respectively. In both cases, the tumour was re-excised after discovery. Both patients are alive and without evidence of disease. In the third patient, the local recurrence was discovered together with pulmonary metastases 15 months after surgery.

### Distant metastasis and management

Twelve patients (35%) developed distant metastases, the majority of which occurred in years one (*n* = 5) and two (*n* = 4). Nine of these 12 patients died. One of the remaining three patients is currently receiving doxorubicin to treat multiple pulmonary metastases. Another patient was diagnosed with a solitary lung lesion 47 months after surgery, which was treated with ablation therapy. However, the patient developed a new lung nodule 7 months later, which has remained stable until the most recent follow-up (10 months). The final patient was diagnosed with a solitary lung lesion 74 months after surgery, which was treated with stereotactic ablative radiotherapy, and is still alive after 57 months with no signs of disease.

### Disease-free survival

Fourteen PASART (41%) patients developed local recurrence, distant metastases or both, with an average time between detection and surgery of 20.0 months (range 2–74 months). DFS at year 1, 2 and 3, and year 4 and 5 were 79.4% (95% CI: 66.9–94.2), 67.6% (95% CI: 53.6–85.3) and 61.3% (95% CI: 46.9–80.3), respectively. Median DFS was not reached (Supplementary Figure 1).

### Overall survival

OS of the combined PASART-trials and IKNL cohort is shown in Supplementary Figure 2. Adding pazopanib to neo-adjuvant radiotherapy did not improve OS (*p* = 0.11). For the PASART cohort OS at year 1, 2, 3, 4, and 5 were 97% (95% CI: 91.5–100), 85.3% (95% CI: 74.2–98.1), 82.4% (95% CI: 70.5–96.2), 79.3% (95% CI: 66.8–94.2), respectively (Supplementary Figure 2).

Cox regression analysis showed statistical significant difference for age, AJCC-stage, FNCLCC grade, tumour subtype, tumour location and resection margin on OS for the whole cohort, as well as borderline statistical significance for sex (Supplementary Table 1). Supplementary Figure 3 displays Kaplan-Meier estimated OS for each variable in the entire cohort. In addition, there was no difference between pCR status and OS for the PASART patients (HR: 1.37, 95% CI: 0.34–5.50).

### Exact matching results

Supplementary Figure 4 shows covariate balance before and after exact matching. After matching 23 PASART (68%) patients and 89 IKNL (18%) patients were included. Patient characteristics for the matched cohort are shown in Table 1. Standardised mean differences of the matched cohort were all within 0.1 for all variable indicating balanced baseline covariates.

**Figure 2:**
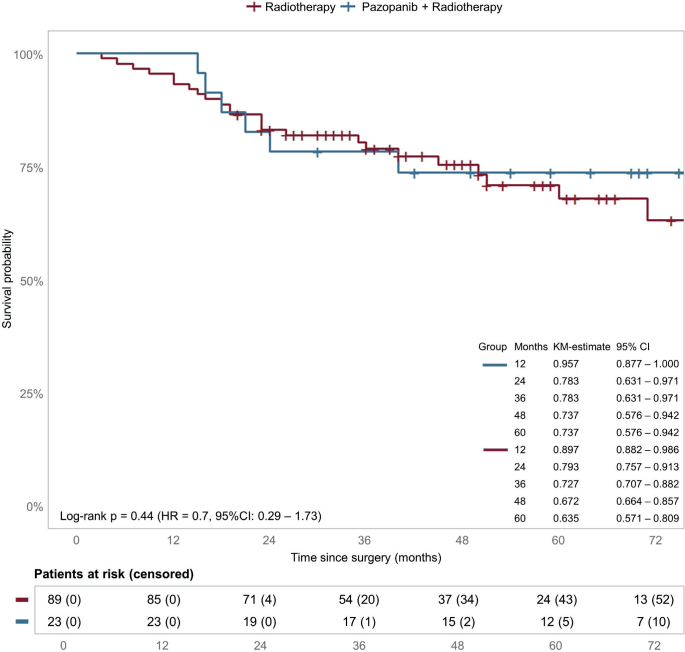
Overall survival matched cohort. Estimated survival and HRs for OS for pazopanib in the matched cohort. Censoring is indicated by tick marks. CI: confidence interval; HR: hazard ratio; KM: Kaplan–Meier.

Kaplan–Meier curve for OS of the matched cohort is shown in Figure 2. Like the whole cohort analysis, adding pazopanib did not improve OS (*p* = 0.44, HR: 0.7, 95% CI: 0.29–1.73).

Alternative matching strategies were conducted for sensitivity analysis, such as testing different ratios, limiting the number of controls per case, and using other matching methods such as coursed exact matching, nearest neighbour matching, optimal pair matching and genetic matching. These different matching strategies produced similar results as exact matching, but either resulted in a lower sample size or less balanced baseline variables (Supplementary Table 2).

## Discussion

In this study the long-time outcomes of the PASART-trials were reported and compared to a control cohort receiving standard treatment during the same period. Taking the limited sample size into account, no significant differences in OS were observed between the pooled, matched and unmatched PASART and IKNL patients, neither an improvement nor a decreased OS. Cox regression analysis revealed that OS was staststically significantly associated with age, AJCC-stage, FNCLCC grade, tumour subtype, tumour location and resection margin, with borderline statistical significance for sex; pCR status was not associated with OS in the PASART patients.

OS in the PASART cohort at 5-year (79.3%) was favourable compared to previous neo-adjuvant radiotherapy studies, which observed 5-year OS between 62.1% and 69.3% [17, 18]. Moreover, while in this study pazopanib was not associated with DFS, the observed DFS percentage at 5 year (61.3%) were favourable compared to earlier neo-adjuvant radiotherapy studies, which reported a 5-year DFS of 50.1% and 65.4% [17, 18]. Therefore, the addition of pazopanib might still be beneficial in STS, even though this study did not show a significant difference.

Conversely, it has been reported that cessation of Vascular Endothelial Growth Factor-targeted therapy might be associated with a rebound and even accelerated outgrowth of tumour cells [19]. Assuming that at least one-third of all locally advanced sarcoma patients harbour occult metastatic disease, permanent pazopanib cessation after completing the pazopanib-based regimen may potentially have a detrimental effect for patients. However, a randomised controlled trial in clear cell renal cell carcinoma did not observed non-inferiority between a drug-free interval strategy and conventional continuation strategy for pazopanib, suggesting that cessation of pazopanib is unlikely to pose major risks [20].

With regard to toxicity of this pazopanib-based regimen, the long-term toxicity profile was acceptable, with a 31% rate of any grade late toxicities, including fibrosis 17.6% (6/34) and localised oedema 5.8% (2/34). These toxicity findings were consistent with previous neo-adjuvant radiotherapy studies, which reported fibrosis rates ranging from 8.5% to 31.5% and oedema rates ranging from 2.2% to 15.1% [18, 21–23].

Although PASART-2 did not meet its primary endpoint of inducing a pCR rate of at least 30%, this pazopanib-based regimen still induced a clinically meaningful pCR rate of 20%, well above phase III trial derived data [8]. While this study was unable to show that pCR rates were associated with survival, pCR was in fact observed to be significantly correlated with a lower recurrence rate and improved OS, and a clinical but not statistically significant improvement in distant recurrence-free survival in previous neo-adjuvant STS studies [18, 24–26]. As a result, increased pCR rates by the addition of pazopanib may still translate into improved survival.

Although the study cohort is prospective in design, the main limitation of this study is a comparison of PASART to a control cohort (IKNL). To compensate for a potential selection bias, we used exact matching in addition to a direct comparison between INKL and PASART. This approach enabled us to also effectively balance baseline factors that are known to influence survival and to partly mimic a randomised control trial. However, the use of matching inherently has limitations, regardless of the matching method used. Most notably, in our study following unmatched patients, the estimation of the effect of pazopanib could be biased and it is unclear to which population the effect applies. Moreover, there may be other relevant variables that were not recorded in our study or did not have a defined prognostic role, resulting in remaining or even increased unbalance. Hence, matching can only mimic randomisation and can only provide an approximation of what a prospective trial may have shown. Therefore, in the ideal setting we would avoid the use of a matched subgroup analysis altogether and instead use multivariable Cox regression for the analysis. However, given the nature of STS, the variables and cohorts were highly heterogeneous, making Cox regression interpretations challenging.

Other limitations of this study are based upon the lack of precise follow-up data of the IKNL cohort. For example, DFS data were missing, making it impossible to determine if adding pazopanib to neo-adjuvant radiotherapy improves DFS. An improved DFS may be especially relevant in STS since there are limited viable therapeutic options in the metastatic setting. Furthermore, since the cause of death for the IKNL cohort was unknown, the OS analysis was not adjusted for disease-related survival, thus underestimating the IKNL patients’ survival and overestimating the beneficial effect of pazopanib. This would be especially relevant when the OS was significantly different. In addition, the relatively small sample size of the combined PASART cohort makes it difficult to draw definitive conclusions on the efficacy of this treatment regimen in STS.

Most of these limitations could be handled by conducting a prospective adequately powered randomised control trial, but given the nature of STS, this will be difficult if not impossible. For example, the discussed PASART-1 and PASART-2 trials were already performed in three tertiary sarcoma referral centres, and accrual took several years to complete. Therefore, another more feasible way to partially overcome these limitations may be by creating better and larger control cohorts to allow for more robust comparisons.

## Future perspectives

Immunotherapy has shown great promise in the treatment of non-metastatic STS. This was recently highlighted by Mowery et al., who demonstrated that the addition of the PD-1 inhibitor prembrolizumab to neo-adjuvant radiotherapy in high-grade undifferentiated pleomorphic sarcoma or dedifferentiated or pleomorphic liposarcoma resulted in a notable 15% improvement in DFS at 2 years [27]. Importantly, the addition of prembrolizumab had acceptable toxicity and did not result increased postoperative complications. Therefore, immunotherapy appears to be a promising new treatment strategy for non-metastatic STS patients that warrants further investigation. In this regard, we are currently conducting the phase 1 SADDRIN-1 trial (NCT05116254), which evaluates the ataxia telangiectasia mutated inhibitor AZD1390 with neo-adjuvant radiotherapy, with or without the PD-L1 inhibitor durvalumab, in patients with newly diagnosed non-metastatic STS.

## Conclusion

In conclusion, this study showed that adding pazopanib to neo-adjuvant radiotherapy was generally well tolerated but did not significantly improve OS when compared to standard treatment. Nevertheless, the pazopanib-based regimen still induced a notable pCR rate of 20%, which is well above data from phase III trials and may have positive effect on survival. Furthermore, the observed survival rates compared favourably to prior neo-adjuvant radiotherapy STS studies. Importantly, a potential accelerated and early manifestation of occult metastatic disease, possible after pazopanib cessation, has not been observed. The potential beneficial effect of this regimen on oncological endpoints remains unclear and warrants further investigation in larger studies.

## Supplementary Material

Long-term survival of participants in the PASART-1 and PASART-2 trials of neo-adjuvant pazopanib and radiotherapy in soft tissue sarcoma

## Data sharing

Data used to supoprt the findings of this study are available from the corresponding author upon request.
