# Supplementary material for: Long-term survival of participants in the PASART-1 and PASART-2 trials of neo-adjuvant pazopanib and radiotherapy in soft tissue sarcoma
Source: Acta Oncol. 2025 Jan 15;64:42333. doi: 10.2340/1651-226X.2025.42333 (PMC11758146; doi:10.2340/1651-226X.2025.42333)
Supplement: Long-term survival of participants in the PASART-1 and PASART-2 trials of neo-adjuvant pazopanib and radiotherapy in soft tissue sarcoma [file AO-64-42333-s1.pdf]

Supplementary material has been published as submitted. It has not been copyedited, or typeset by Acta Oncologica

Supplementary material

Long-term survival of participants in the PASART-1 and PASART-2 trials of neo-adjuvant pazopanib and radiotherapy in soft tissue sarcoma

Tables and Figures

- Supplementary Table 1. Hazard Ratio (HR) and 95% confidence interval (CI) for Overall Survival estimated with a univariate Cox Regression model for pooled INKL and PASART cohorts, and number of patients and events for each variable and cohort
- Supplementary Table 2. Hazard Ratios for overall survival matching methods
- Supplementary Figure 1. Disease-Free Survival PASART-1 and PASART-2
- Supplementary Figure 2. Overall survival PASART-trials and IKNL
- Supplementary Figure 3. Estimated overall survival
- Supplementary Figure 4. Matching covariate balance

Supplementary Table 1. Hazard Ratio (HR) and 95% confidence interval (CI) for Overall Survival estimated with a univariate Cox Regression model for pooled INKL and PASART cohorts, and number of patients and events for each variable and cohort

	No. Patients (events)		Cox regression	
Variables	PASART	IKNL	HR	95% CI
Sex				
Male (ref.)	21 (7)	284 (106)	-	-
Female	11 (2)	203 (62)	0.74	0.54 – 1.00
Age at diagnosis (years)	-	-	1.03	1.02 – 1.04
Age in groups				
<50 years (ref.)	10 (4)	124 (23)	-	-
≥50 - <65 years	14 (4)	148 (46)	1.50	0.94 – 2.40
≥65 years	10 (1)	215 (99)	2.69	1.76 – 4.11
AJCC-stage				
Stage I	3 (1)	-	1.28	0.18 – 9.23
Stage II (ref.)	18 (2)	231 (53)	-	-
Stage III	13 (6)	251 (111)	2.50	1.81 – 3.44
Tumour size				
<5cm (ref.)	4 (0)	65 (17)	-	-
≥5cm	30 (9)	417 (147)	1.51	0.91 – 2.45
Tumour depth				
Superficial (ref.)	4 (1)	180 (56)	-	-
Deep	30 (8)	209 (86)	1.36	0.97 – 1.89
FNCLCC grade				
Grade I/II (ref.)	20 (3)	192 (44)	-	-
Grade III	14 (6)	295 (124)	2.40	1.72 – 3.35
Histological subtype				
USTS (ref.)	15 (5)	130 (57)	-	-
Myxofibrosarcoma	9 (1)	84 (31)	0.79	0.51 – 1.21
MLS	1 (1)	110 (14)	0.25	0.14 – 0.45
PLS	1 (0)	19 (8)	0.95	0.46 – 1.99
MPNST	2 (0)	20 (7)	0.73	0.34 – 1.60
RMS	2 (1)	3 (1)	0.78	0.19 – 3.18
Synovial sarcoma	2 (1)	19 (6)	0.78	0.36 – 1.71
Epithelioid sarcoma	1 (0)	5 (2)	0.73	0.18 – 2.99
DDLPS	-	26 (7)	0.65	0.30 – 1.42
Liposarcoma NOS	-	7 (4)	2.11	0.76 – 5.82
LMS	-	33 (17)	1.18	0.69 – 2.02
Other/Unknown sarcoma	1 (0)	31 (14)	0.93	0.52 – 1.67
Tumour site				
Extremity (ref.)	26 (6)	388 (119)	-	-
Trunk	8 (3)	99 (49)	1.75	1.26 – 2.41
Resection margin				
R0 (ref.)	32 (9)	369 (109)	-	-
R1	2 (0)	67 (34)	2.01	1.37 – 2.94
R2	-	5 (5)	7.09	2.88 – 17.45

AJCC, American Joint Committee on Cancer; DDLPS, dedifferentiated liposarcoma; FNCLCC, Fédération Nationale des Centres de Lutte Contre le Cancer; IQR, interquartile range; pCR, pathologic complete response; MPNST, Malignant Peripheral Nerve Sheath Tumour; MLS, Myxoid Liposarcoma; USTS, Undifferentiated Soft Tissue Sarcoma; PLS , Pleomorphic Liposarcoma; RMS, Rhabdomyosarcoma; LMS, Leiomyosarcoma (excluding skin); CI, Confidence interval; HRs, hazard ratios; KM, Kaplan-Meijer.

Supplementary Table 2. Hazard Ratios for overall survival matching methods

Matching Method	Ratio	No. of Patients		Cox regression	
		PASART	IKNL	HR	95% CI
None, Direct Comparison	None	34	487	0.58	0.30 – 1.13
Exact Matching	None	23	89	0.70	0.29 – 1.73
Coursed Exact Matching	1:1	20	20	0.64	0.19 – 2.15
Nearest Neighbour Matching	1:2	34	68	0.47	0.22 – 0.99
Optimal Pair Matching	1:2	34	68	0.50	0.23 – 1.07
Genetic Matching	1:2	34	68	0.62	0.27 – 1.39

Supplementary Table 1. Cox Model for Overall survival by considering different matching methods, CI, confidence Interval; HRs, hazard ratios; OS, overall survival; No., number.

Supplementary Figure 1. Disease-Free Survival PASART-1 and PASART-2

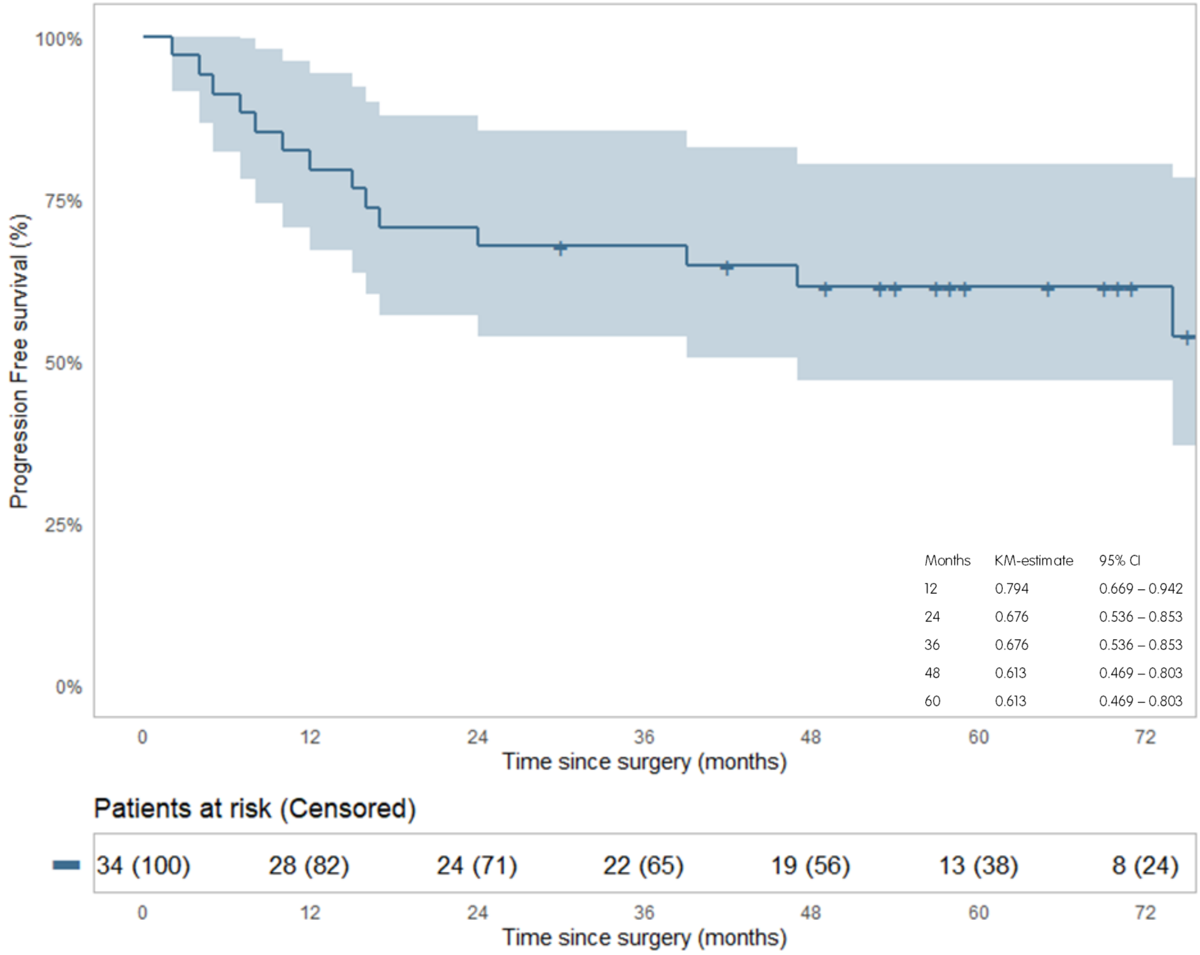

Supplementary Figure 1. OS for PASART-1 and PASART-2. The shaded area represents the confidence interval. Censoring is indicated by tick marks.
CI: confidence interval; KM, Kaplan-Meijer.

Supplementary Figure 2. Overall survival PASART-trials and IKNL

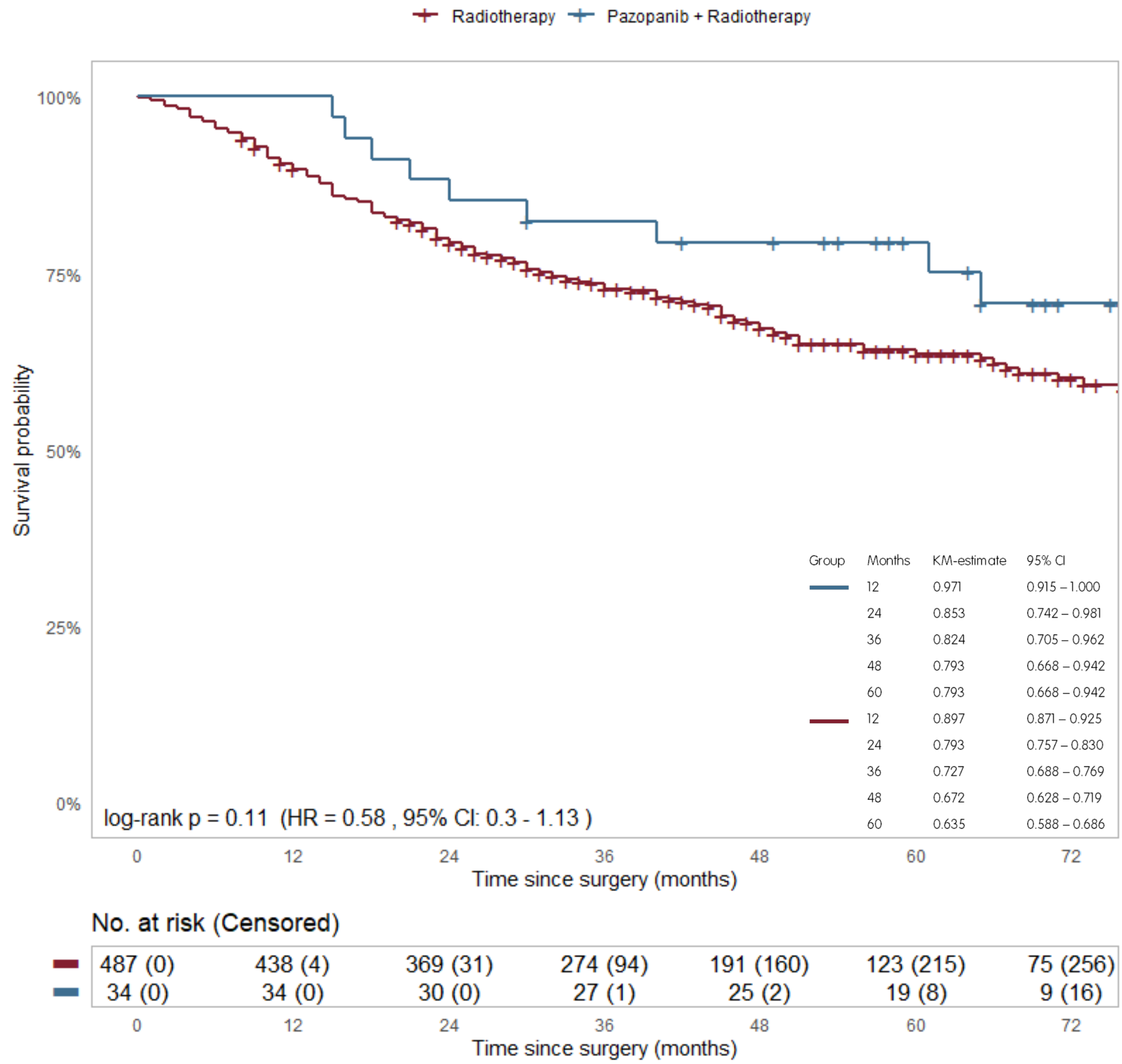

Supplementary Figure 2. Estimated survival and HRs for OS for pazopanib. Censoring is indicated by tick marks. CI, Confidence interval; HR, hazard ratio; KM, Kaplan-Meijer.

Supplementary Figure 3. Estimated overall survival

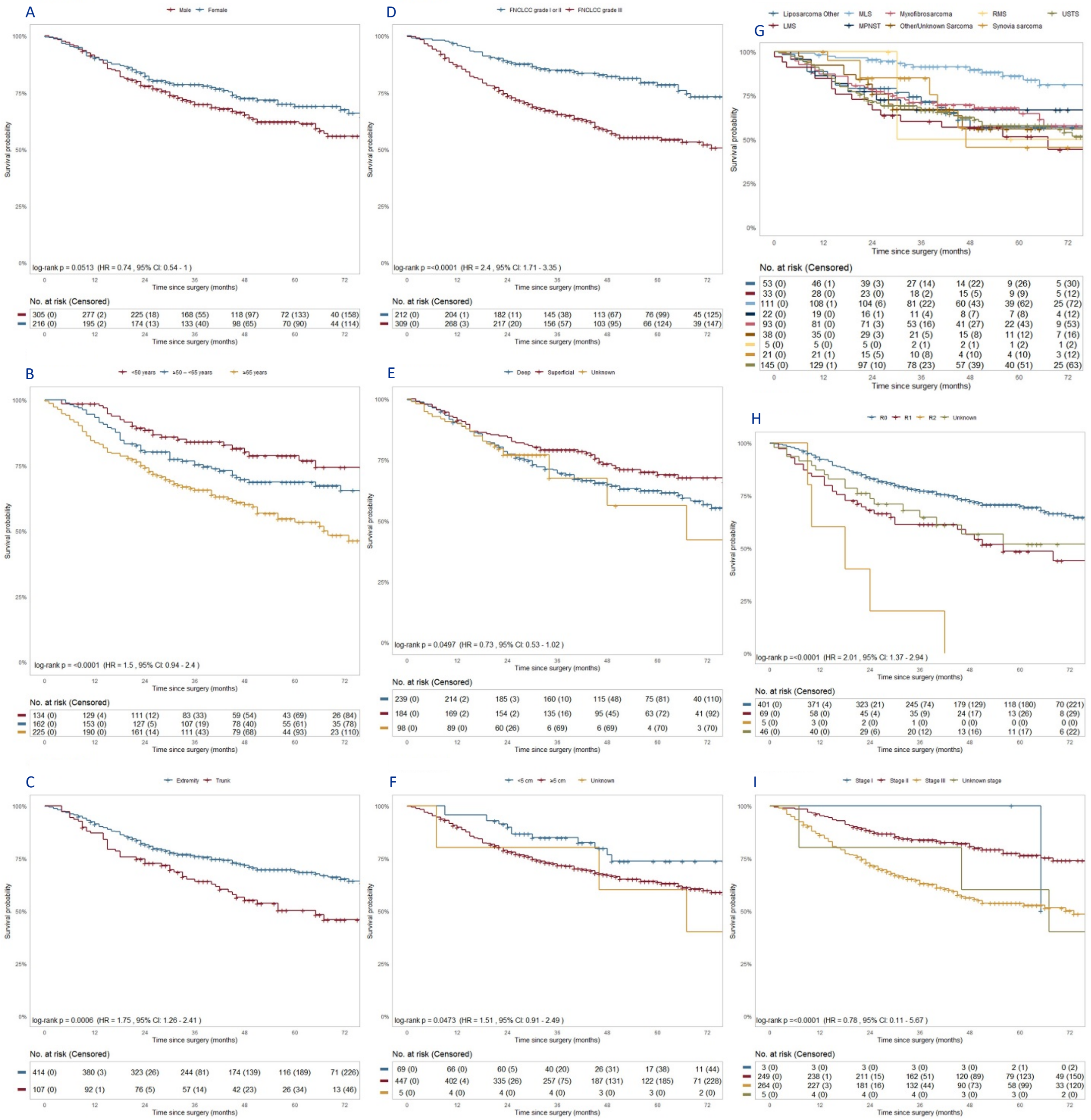

Supplementary Figure 3. Estimated survival and hazard ratio (HR) for overall survival (OS) for: age (figure A), sex (figure B), tumour site (figure C), FNCLCC grade (figure D), tumour depth (figure E), tumour size (figure F), histological subtype (figure G), resection margin (figure H) and AJCC-stage (figure I). All P values indicated on the Kaplan-Meier curves correspond to log-rank test. HR and 95% CI are estimated with a Cox regression model. Censoring is indicated by tick marks.
AJCC, American Joint Committee on Cancer stage; FNCLCC, Fédération Nationale des Centres de Lutte Contre le Cancer;

Supplementary Figure 4. Matching covariate balance

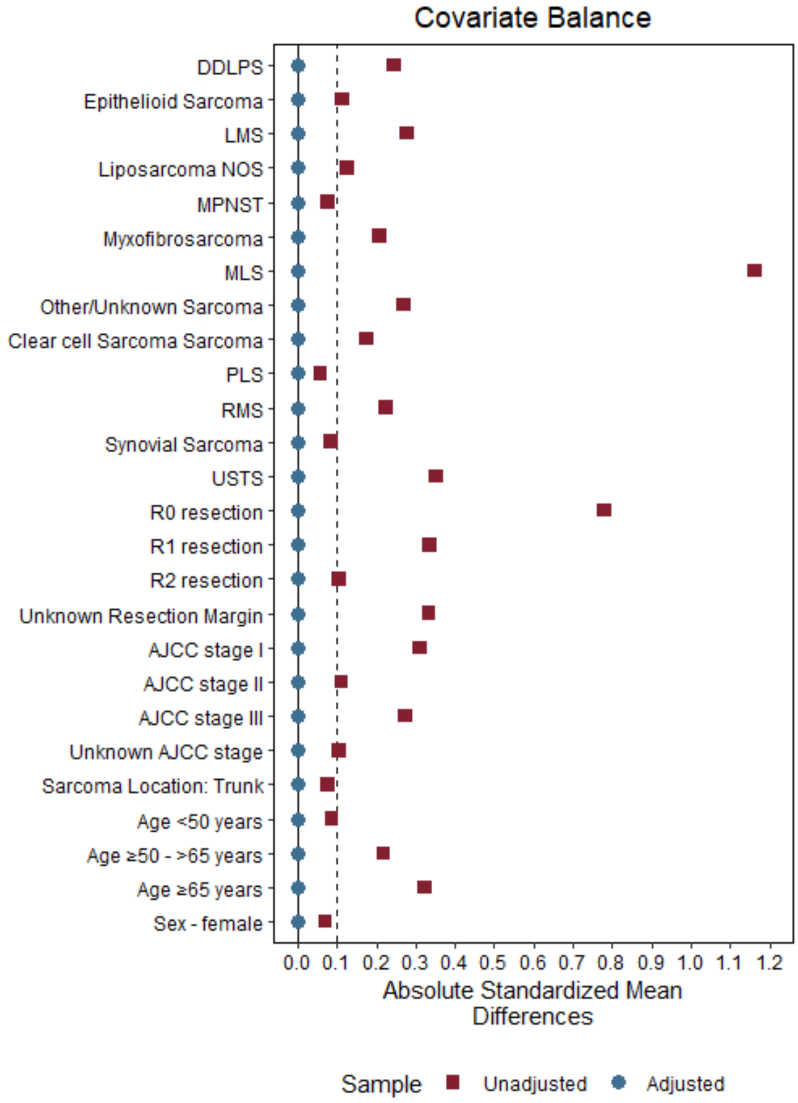

Supplementary Figure 4. Matching covariate balance,
AJCC, American Joint Committee on Cancer; DDLPS, dedifferentiated liposarcoma;; MPNST, Malignant Peripheral Nerve Sheath Tumour; MLS, Myxoid Liposarcoma; UPS, Undifferentiated Pleiomorphic Sarcoma; PLS , Pleomorphic Liposarcoma; RMS, Rhabdomyosarcoma; LMS, Leiomyosarcoma (excluding skin);
